# Genetic Effects of GA-Responsive Dwarfing Gene *Rht13* on Plant Height, Peduncle Length, Internodal Length and Grain Yield of Wheat under Drought Stress

**DOI:** 10.3390/genes14030699

**Published:** 2023-03-11

**Authors:** Muhammad Arslan Khalid, Zulfiqar Ali, Muhammad Hammad Nadeem Tahir, Abdul Ghaffar, Javed Ahmad

**Affiliations:** 1 Institute of Plant Breeding and Biotechnology, MNS University of Agriculture, Multan 66000, Pakistan; 2 Department of Plant Breeding and Genetics, University of Agriculture Faisalabad, Faisalabad 38000, Pakistan; 3 Programs and Projects Department, Islamic Organization for Food Security, Astana 019900, Kazakhstan; 4 Department of Agronomy, MNS University of Agriculture, Multan 66000, Pakistan; 5 Wheat Research Institute, Ayub Agricultural Research Institute Faisalabad, Faisalabad 38000, Pakistan

**Keywords:** wheat, dwarfing genes, reduced height, line × tester, comparative expression analysis, drought tolerance

## Abstract

Reduction in plant height is generally associated with an increase in lodging resistance, drought tolerance and grain yield of wheat worldwide. Historically, a significant increase in grain yield was observed through the introduction of semi-dwarf wheat varieties utilizing the gibberellic acid-insensitive *Rht* genes (*Rht1* or *Rht2*). The gibberellic acid sensitive (GA-sensitive) reduced height (*Rht*) genes are available that are alternatives to gibberellic acid insensitive (GA-insensitive) *Rht* genes, having a neutral effect on coleoptile length seedling vigor suggesting their potential in using alone or in combination with GA-insensitive *Rht* genes to improve grain yield and drought tolerance in wheat. This study was conducted to evaluate parents and F_1_ crosses under drought stress. The crossing was done using line × tester mating design, comprising eight lines and five testers having different GA-sensitive and GA-insensitive *Rht* genes. Parents and F_1_ crosses were sown in the field under RCBD with three replications in normal and drought stress. Data were recorded for morpho-physiological traits. The mean comparison showed significant differences among parents and hybrids for most of the studies’ traits. The general combining ability showed that line 1 is the good general combiner for days to heading, lodging (%), plant height, peduncle length, internodal length and days to maturity under normal conditions while L5 was the good general cobiner for chlorophyll contents and stomatal conductance both under normal and drought stress. The spcaicfic combing ability estimases showed that the cross L1 × T1 was best for days to heading, lodging (%), plant height and internodal length both under normal and drought stress. F_1_ hybrids showed a significant reduction in plant height (18–25%), peduncle length (20–28%) and increased grain yield (15–18%) under drought stress. Expression analysis showed upregulation of *Rht13* at the middle part of the peduncle internode under drought stress. From the expression analysis, five crosses were selected, and their segregating population was raised and space-plated. *Rht13* genes reduced plant height (−30 to −45%), peduncle length (−30 to −53%), peduncle internode length (−28% to −48%), increased spike length (+20% to +50%), number of grains per spike (+17 to +26%) and grain yield per plant (+29% to +50%) compared to *Rht1* gene. These results suggested the possibility of using the GA-sensitive *Rht13* gene for the development of high-yielding and drought-tolerant wheat varieties.

## 1. Introduction

Plant breeders aim to find new genetic recombination to produce high-yielding and superior genotypes. This can be achieved by crossing the good general combiner parental genotypes and selecting the transgressive segregants from the obtained hybrids for the grain yield and its associated traits [1]. The knowledge about the combining ability is most important as it evaluates the differences among various genotypes and estimates the extent and nature of gene action for a specific trait. Combining ability helps in the selection of parents and crosses for the final decision of breeding method to be used for the improvement of the trait [2]. One of the most essential analyses for estimating the general combining ability (GCA) of the parental lines and the specific combining ability (SCA) of the F_1_ hybrids is line × tester [3]. This analysis provides the breeding material combining ability effects and information about the genetic mechanism which controls various yield-determining traits [2]. Understanding SCA and GCA for yield contributing traits is essential for plant breeders to improve drought tolerance in wheat [2]. These aid the breeders in the selection of the desired parental material for developing desired hybrids, especially in cross-pollinated crops [4]. Many researchers worked on the combining ability for determining the gene action of the wheat breeding population by using line × tester analysis for various yield-contributing traits [4,5]. So, this analysis is the most suitable way to select promising parental lines and the best cross combination.

To produce high-yielding, drought-tolerant varieties, a complete understanding of different morphophysiological characters like chlorophyll contents [6], stomatal conductance [7], plant height [6], spike length [6], number of grains/spike [6] and grain yield/plant [8] is essential. The selection of parental genotypes is important to develop drought stress tolerance [7]. The selected genotypes not only have the desired traits but have the ability to produce superior hybrids by crossing with another suitable parent. Combining ability analysis provides the most suitable information for the selection of parental lines. Significant GCA and SCA effects for the number of grains/spike [8], tillers/plant [9], flag leaf area [10] and grain yield/plant [11] have been reported.

The utilization of semi-dwarf wheat varieties extensively resulted in increased world wheat yield during the 1960s and 1970s, which led to the green revolution [12]. The two main “green revolution” genes, i.e., *Rht-B1b* and *Rht-D1b*, encode DELLA protein-altered forms, which act as key repressors for the signaling pathway of gibberellin [13]. These dwarfing genes are well known to reduce cell size in coleoptiles, culms and leaves of wheat. As compared to standard height genotypes (*Rht*-*B1a* and *Rht-D1a*) of wheat, GA-insensitive wheat is poor in the case of leaf area development [14]. The alternative height-reducing genes (GA-sensitive) *Rht8* and *Rht13* shorten plant height and enhanced grain yield [14]. The GA-sensitive (*Rht13*) is the most important dwarfing gene that controls the plant height of wheat without influencing the seedling growth, grain number and grain yield [15,16]. *Rht13b* dwarfing allele reduces plant height by up to 34% compared to *Rht13a*, *Rht1* and *Rht2*, depending on the growing conditions and genetic background [17,18]. Limited work was done on the effect of the *Rht13* gene on wheat growth and yield under drought stress.

In the present study, line × tester mating design was used to cross wheat genotypes. The contrasting wheat genotypes were hybridized to incorporate GA-sensitive *Rht* genes and improve drought tolerance and grain yield. This study aimed to identify genes involved in drought stress and create high-yielding, drought-tolerant wheat breeding material with *Rht13* alone or in combinations with other GA-insensitive or sensitive *Rht* genes. So, this breeding strategy was designed to develop high-yielding and drought-tolerant wheat breeding material having the *Rht13* gene alone or in combination with other GA-insensitive or sensitive *Rht* genes.

## 2. Materials and Methods

This study comprised 13 wheat genotypes, including eight lines and five testers. The list of lines and testers is presented in Table 1, and the list of crosses is presented in Table 2. The genotypes (eight lines and five testers) were selected for hybridization in the line × tester matting design [19]. The hybrids, along with their parents, were sown in three replications under RCBD under normal (03 irrigations) and drought stress (01 irrigation); each irrigation was 120 mm. The data were recorded for days to heading (days) from sowing to 50% of heading, lodging (%) calculated by the formula (Lodging% = %plot area lodged × angle of lodging/90) [20], at physiological maturity plant height (cm), peduncle length (cm), internodal length (cm), stem diameter (mm) was calculated by vernier caliper from top, middle and bottom, then the average value of the stem diameter was calculated, days to maturity (days) was recorded from days from germination to the days to physiological maturity, spike length (cm), number of grains/spike, 1000-grain weight (g), grain yield/plant (g), chlorophyll contents (mg/g dry weight) were recorded from the base, the mid and upper part of the flag leaf with the SPAD 502 Plus Chlorophyll Meter (Spectrum Technologies Inc., Aurora, IL, USA) and then average value for chlorophyll contents was calculated. The stomatal conductance was estimated by using CIRAS-3 Portable CO_2_/H_2_O Gas Analysis System, PP Systems, Amesbury, MA, USA. External air was scrubbed of CO_2_ and mixed with a supply of pure CO_2_ to create a reference concentration of 390–400 μmol m^−2^ s^−1^. The CO_2_ concentration was maintained at a constant level by using ambient CO_2_ and H_2_O. The system was equipped with a leaf cuvette that exposed 4.5 cm^2^ of leaf area. The mid-portion of the top three leaves of the selected plant was kept in a leaf chamber during the measurements. Variations between lines, testers and F_1_ hybrids were estimated by the analysis of variance (ANOVA) by using the Agricolae R software 3.6.3 package [9]. Furthermore, correlation [10] and principal component analysis (PCA) were also performed for yield contributing traits [11].

### 2.1. Comparative Expression Analysis of Rht13 Gene in F_1_ Hybrids

This experiment comprised 40 F_1_ hybrids which were grown in the field under RCBD with three replications under normal (03 irrigations) and drought stress (01 irrigation); each irrigation was 120 mm. The samples were taken from the middle part of the peduncle internode at the booting stage [12]. Isolation of total RNA was carried out using the TRIzol^®^ method (https://www.abcam.com/protocols/rna-isolation-protocol-cells-in-culture accessed on 11 November 2021) and complementary DNA (cDNA) synthesis, and Semi qPCR was performed for expression analysis of *Rht* genes [13].

The 5µL RNA (500 ng) was used for the synthesis of cDNA. For cDNA synthesis, take 5 µL RNA in the PCR tube, add 6.5 µL nuclease-free water and 1 µL oligo dt to make the total volume 12.5 µL. Then centrifuge the sample for 2 min at 50,000× *g* rpm so that all reagents are mixed. Run the samples in the thermocycler at 65 °C for 5 min. When the run is completed, then take the sample and put the ice on for 5 min to lower the temperature. Then add Riblock RNase inhibitor (0.5 μL), 5× reaction buffer (4 μL), dNTP’s (2 μL of 10 mM concentration) and Revertaid Reverse Transcriptase (1 μL). Now the total volume is 20 μL. The centrifuge samples must be at 5000× *g* rpm for 2 min for mixing of reagents. Run the samples in a thermocycler at 42 °C for 60 min, 70 °C for 10 min, 10 °C for 15 min and for final termination at 72 °C for 1 min. Store the synthesized cDNA at −70 °C.

Twenty μL final volume of PCR reactions containing 12.8 μL ddH_2_O, 1 μL cDNA (20 ng/μL) as reported by Maryam et al. [14], 0.4 μL of each primer (10 μM) (list of primers are given in Table 3), 2 μL rTaq buffer (10×), 1.6 μL MgCl_2_ (25 mM), 1.6 μL dNTPs (10 mM), and 0.2 μL rTaq DNA polymerase (5 U/μL) (Takara Bio Inc.; Shiga, Japan). PCR was performed in a thermo-cycler (T100 Thermal Cycler, Bio-Rad Laboratories, Inc., Hercules, CA, USA) set with an initial denaturation temperature of 94 °C for 3 min, 35 cycles of denaturation at 94 °C for 30 s, annealing at 50–60 °C for 30 s and extension at 72 °C for 60 s/kb of amplicon followed by a final extension at 72 °C for 3 min/kb of the amplicon. Resolve amplicons on 1% agarose gel [14,15]. The band intensity was identified on ImageJ software (https://imagej.nih.gov/ij/download.html accessed on 6 December 2021) [16].

### 2.2. Field Evaluation of Segregating Population

From the results of comparative expression analysis (Figure 7), five F_1_ hybrids were selected on the basis of upregulation of the *Rht13* gene under drought stress in the middle part of the peduncle internode (Figure 7). The F_1_ plants were raised during 2019–2020 in the field at MNS University of Agriculture, Multan, and selfed to produce F_2_. A total of five F_2_ populations (Table 4) were spaced planted (0.17 m space) in 3–5 cm sowing depth, keeping the plot size 1.2 × 5 m with the seeding rate of 200 seeds per meter square. The recommended nutrient application was made accordingly. The herbicide applications were made to keep the plots weed-free. Data from the 20 guarded plants were recorded at physiological maturity. The data were collected for plant height (cm), peduncle length (cm), internodal length (cm), ear length (cm), number of tillers per plant, number of spikelets per spike, number of grains per spike and grain yield per plant (g), separately. The data collected were statistically analyzed by following the formula outlined by [9]. Furthermore, PCA was calculated for yield traits [11]. The mean comparison was calculated by using Tukey’s Honesty Significant Difference (HSD) test at 0.05. The statistical analysis was carried out using Agricolae R software [9].

## 3. Results

Analysis of variance for the various morphological and physiological traits of parents and F_1_ hybrids under normal and drought stress is shown in Table 5 and Table 6, respectively. Under normal conditions, significant differences were observed for all the studied traits among treatments, parents and parents vs. crosses. The lines and tester differed insignificantly for all the studied traits except for days to heading, 1000-grain weight, grain yield/plant, and stomatal conductance, which differed significantly among tester, while spike length and the number of grains/spike differed significantly among the testers. Under drought stress in the field, it was recorded that treatments, parents, parents vs. crosses, crosses, lines and lines × tester differed significantly for all the studied traits except for days to heading, stem diameter and days to maturity which differed insignificantly for parents vs. crosses. While lines and testers showed insignificant differences for all the studied traits except for days to heading, grain yield/plant and stomatal conductance, which differed significantly among lines, while days to heading, spike length and number of grains/spike differed among the tester.

The general combining ability (GCA), the proportional contribution of lines, tester and their interaction (lines × tester) to total variance under normal and drought stress in the field is presented in Table 7 and Table 8, respectively. Under normal conditions, results showed that in the case of morphological traits, L1 showed significant positive effects on the days to heading, lodging (%), plant height, peduncle length, internodal length and days to maturity, while L2 showed significant positive effects for spike length, 1000-grain weight and grain yield/plant and thus recommended as a good general combiner for these traits. At the same time, the L5 showed a significant positive effect on the chlorophyll contents and stomatal conductance and was declared a good combiner for these traits. So, these lines may be used for the improvement of yield and physiological traits. It was observed that the contribution of lines × tester was greater for all the studied traits determining DTH, LOD, PH, PL, INTL, SD, DTM, SL, NGS, TGW, GYP, CC and SC, showing 51.08%, 71.79%, 85.07%, 67.98%, 86.77%, 65.26%, 72.70%, 49.97%, 58.49%, 50.47%, 57.05%, 86.73% and 57.88%, respectively, proportional to the total variance (Table 7). Among lines and tester, the lines contribute more for DTH, LOD, PL, SD, DTM, TGW, GYP, CC and SC, showing 38.3%, 17.95%, 16.09%, 29.76%, 24.28%, 39.14%, 35.89%, 10.02% and 33.25%, respectively proportion to the total variance. Moreover, the tester showed more contribution than lines in the case of PH, INTL, SL and NGS, showing 10.85%, 8.76%, 28.58% and 27.29%, respectively.

Under drought stress in the field, it was observed that L1 showed better combining ability for days to heading, lodging (%), plant height and internodal length, while L2 showed significant effects on peduncle length, stem diameter, 1000-grain weight and grain yield. In the case of physiological traits, L5 showed a significant effect on chlorophyll contents and stomatal conductance. Lines showing positive GCA effects were declared the best general combiner for these traits and may be used in breeding programs for the improvement of these traits. It was found that the contribution of all the studied traits was greater to the total variance except for days to heading in which lines showed more contribution determining DTH, LOD, PH, PL, INTL, SD, DTM, SL, NGS, TGW, GYP, CC and SC showing 43.08%, 71.79%, 85.07%, 63.24%, 82.07%, 65.92%, 77.75%, 46.94%, 54.49%, 59.82%, 55.12%, 85.01% and 52.01%, respectively (Table 8). Among the lines and tester, the maximum contribution was recorded from lines in the case of DTH, LOD, PL, SD, DTM, SL, TGW, GYP, CC and SC, showing 43.08%, 17.95%, 18.73%, 28.66%, 16.45%, 23.87%, 28.75%, 37.32%, 10.89% and 37.52%. The testers showed maximum contribution for INTL and NGS, showing 11.35% and 28.46%, respectively.

The results of SCA effects under normal and drought stress in the field are mentioned in Table 9 and Table 10, respectively. Under normal conditions, results exhibited that L1 × T1 was best for days to heading, lodging (%), plant height and internodal length. The cross L4 × T2 was best for stem diameter, L7 × T5 for days to maturity, L4 × T1 for spike length and number of grain/spike, L2 × T2 for 1000-grain weight and L6 × T1 for grain yield/plant, chlorophyll contents and stomatal conductance. The crosses showing significant positive SCA estimates are recommended as good specific combiners for these traits under normal conditions. Under drought stress in the field, it was observed that the cross L1 × T1 was best for days to heading, lodging (%), plant height, peduncle length and internodal length. The cross L4 × T2 was best for stem diameter, L7 × T5 for days to maturity, L4 × T1 for spike length and number of grains/spike, L6 × T3 for 1000-grain weight, and L6 × T1 was best for grain yield/plant, chlorophyll contents and stomatal conductance. The genetic components of lines, tester and their interactrion also showed significance of covariance, heritability and the value of additive varice was more compared to the genetic variance suggesting that the hybrid breeding is not suitable for imrpvement of these traits (Table 11 and Table 12) The crosses showing high and significant SCA estimates for these traits are recommended as good specific combiners and best crosses.

**Figure 1:**
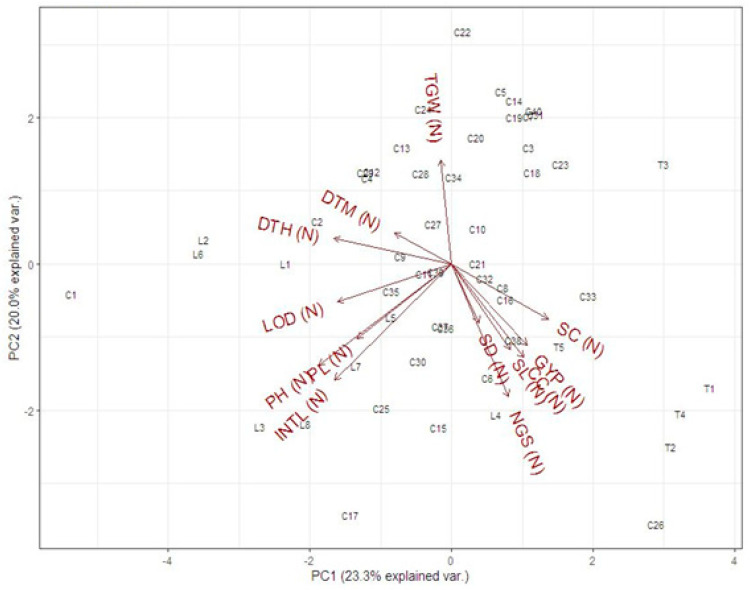
Biplot display of physiological and yield contributing traits viz. days to heading (DTH), lodging (LOD, plant height (PH), peduncle length (PL), peduncle internodal length (INTL), stem diameter (SD), days to maturity (DTM), spike length (SL), number of grains/spike (NGS), 1000-grain weight (GW), grain yield/plant (GY), chlorophyll contents (CC) and stomata conductance (SC) of parents and crosses under normal (N) in the field. Arrows depict the correlation among these traits, and the positive and negative shows the positive/negative correlation.

**Figure 2:**
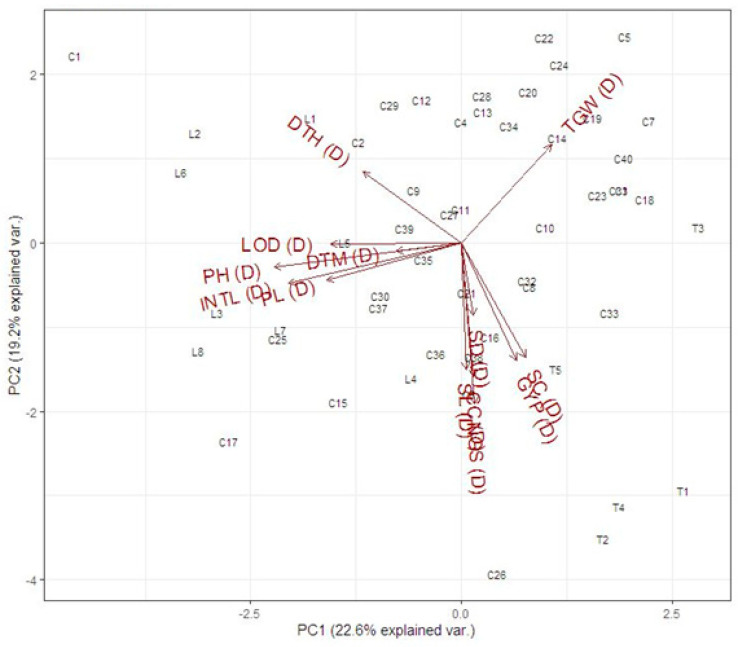
Biplot analysis for yield traits viz. days to heading (DTH), lodging (LOD, plant height (PH), peduncle length (PL), peduncle internodal length (INTL), stem diameter (SD), days to maturity (DTM), spike length (SL), number of grains/spike (NGS), 1000-grain weight (GW), grain yield/plant (GY), chlorophyll contents (CC) and stomata conductance (SC) of parents and crosses under drought (D) stress in the field. Arrows depict the correlation among these traits, and the positive and negative shows the positive/negative correlation.

The biplot analysis for the studied traits under normal and drought stress in the field is presented in Figure 1 and Figure 2, respectively. Under normal conditions, the biplot analysis showed a 23.3% variation for PC1 and a 20.0% variation for PC2 (Figure 1). The results showed that the cross C22 showed maximum value for 1000-grain weight, and line L2 and L6 for days to heading and days to maturity. Moreover, the cross C23, C15 and tester T3 showed less value for these traits. The Cross C1, C-17, C-26 and tester T1, T4 and T2 showed the best value for plant height, peduncle length, internodal length, lodging (%), number of grains/spike, stem diameter, grain yield/plant, chlorophyll content and stomatal conductance. The cross C2, C5 and C14 showed poor performance for these traits. Under drought stress in the field, the PC1 and PC2 showed 22.6% and 19.2% of total variability, respectively (Figure 2). It was observed that the cross C1, C5 and C22 was best for days to heading and 1000-grain weight. The cross C17 and T1 was poor for 1000-grain weight and days to heading. The crosses C17, C26, tester T2, T4 and lines L8 were best for plant height, days to maturity, internodal length, peduncle length, spike length, stem diameter, number of grains/spike, grain yield/plant, chlorophyll contents and stomatal conductance. The line L2, L6, cross C24, C7 and tester T3 showed poor performance for plant height, days to maturity, internodal length, peduncle length, spike length, stem diameter, number of grains/spike, grain yield/plant, chlorophyll contents and stomatal conductance. It was suggested that the lines, tester and crossing, showing the best performance for these traits, are selected for further studies.

**Figure 3:**
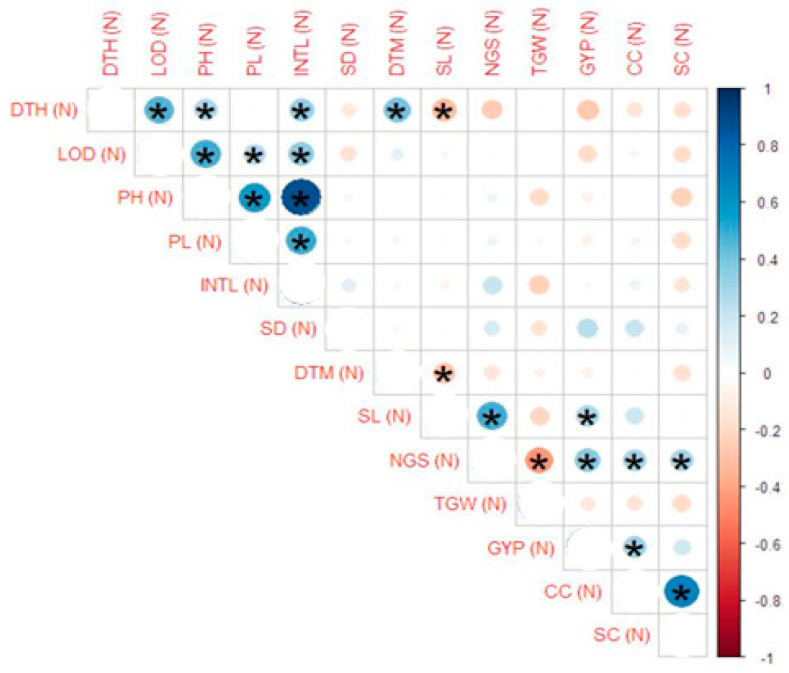
Correlation estimates of yield traits viz. days to heading (DTH), lodging (LOD, plant height (PH), peduncle length (PL), internodal length (INTL), stem diameter (SD), days to maturity (DTM), spike length (SL), number of grains/spike (NGS), 1000-grain weight (GW), grain yield/plant (GY), chlorophyll contents (CC) and stomatal conductance (SC) of parents and crosses under normal conditions in the field. Blue shade shows positive association, and the light pink shade depicts the negative correlation. The stars (*) on these shades shows the significance (*p* ≤ 0.05) of correlation. The circle size showed the degree of association among the traits. The greater the size, the stronger the association will be.

**Figure 4:**
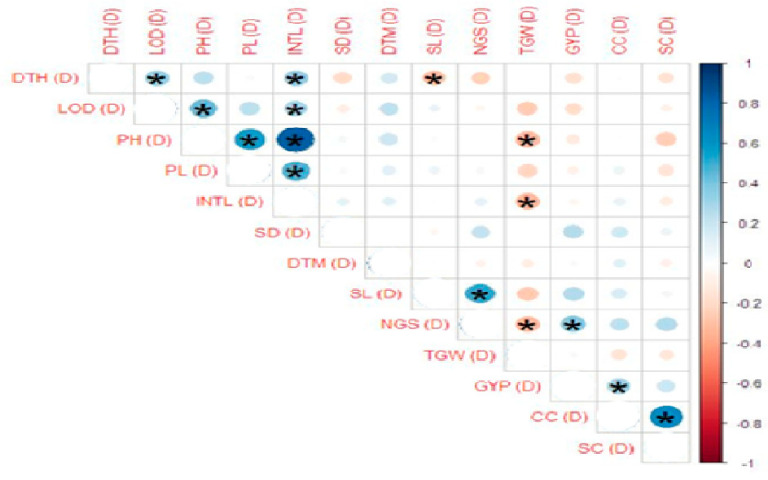
Correlation estimates of yield traits viz. days to heading (DTH), lodging (LOD), plant height (PH), peduncle length (PL), internodal length (INTL), stem diameter (SD), days to maturity (DTM), spike length (SL), number of grains/spike (NGS), 1000-grain weight (GW), grain yield/plant (GY), chlorophyll contents (CC) and stomatal conductance (SC) of parents and crosses under drought stress conditions in the field. Blue shade shows positive association, and the light pink shade depicts a negative correlation. The stars (*) on these shades show the significance (*p* ≤ 0.05) of correlation. The circle size showed the degree of association among the traits. The greater the size, the stronger the association will be.

Correlation estimates for the studied traits showed that under normal conditions, plant height, days to heading, internodal length and lodging (%) had significant positive correlation, while plant height also showed a significant negative correlation with spike length. Plant height is also positively correlated with peduncle length and internodal length. The number of grains/spike had a significant negative correlation with 1000-grain weight, while it was positively correlated with grain yield/plant, chlorophyll contents and stomatal conductance. Grain yield/plant showed a positive correlation with chlorophyll contents. Chlorophyll contents were positively correlated with stomatal conductance (Figure 3). Under drought stress in the field, days to heading showed a significant positive correlation with lodging (%) and internodal length, while it had a significant negative correlation with spike length. Lodging (%) is positively correlated with plant height and internodal length. Plant height showed a positive correlation with peduncle length and internodal length while a negative correlation with 1000-grain weight. Internodal length is also negatively correlated with 1000-grain weight. The number of grains/spike showed a negative correlation with 1000-grain weight and a positive correlation with grain yield/plant. Grain yield/plant showed a positive correlation with chlorophyll contents. Chlorophyll contents were positively correlated with stomatal conductance (Figure 4). It was suggested from the obtained results that the traits showing a positive correlation with grain yield may be selected for yield improvement, while careful selection should be made for those traits having a negative correlation with grain yield.

### 3.1. Comparative Expression Analysis of Rht13 in F_1_ Hybrids

**Figure 5:**
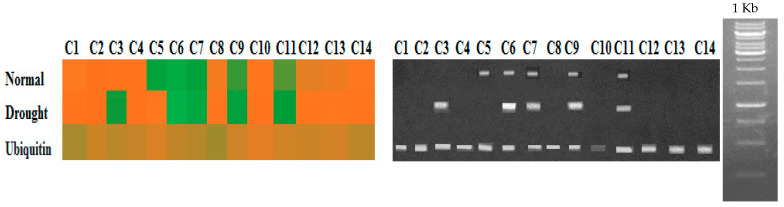
Comparative Expression Analysis of *Rht13* gene in cDNA from of crosses (C) under normal (N) and drought (D) stress. The color in the heat map shows the lowest expression level (orange) and highest expression level (green), the ubiquitin is the internal control. The side 1 Kb ladder showed that the *Rht13* gene has the band size of 1089 bp.

**Figure 6:**
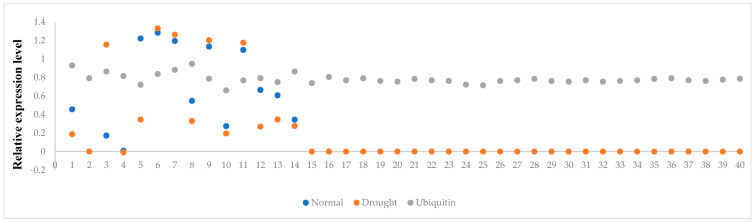
Relative expression level of *Rht13* gene among 40 crosses (C) at the middle part of peduncle node under both normal and drought stress. Ubiquitin (UBQ5) is used as an internal control.

The expression analysis of the *Rht13* gene was carried out at the middle part of the peduncle node in the 40 F_1_ hybrids to check the function of *Rht13* in the hybrids under both normal and drought. The results revealed variable expression of *Rht13* among the hybrids both under normal and drought (Figure 5). It was observed that **under normal conditions,** the crosses C5, C6 and C7, showed two-fold upregulation of *Rht13* in the middle part of the peduncle node. **Under drought stress,** C6, C7, C9 and C11 showed two-fold upregulation of *Rht13* in the middle part of the peduncle internode. Moreover, *Rht13* did not express in the rest of the hybrids. The analysis of the relative expression level (Figure 6) showed that Cross 6 showed maximum expression of *Rht13* followed by C7, and the lowest expression was observed in C2 from the middle part of the peduncle node under both normal and drought stress.

### 3.2. Field Evaluation of Segregating Population

**Figure 7:**
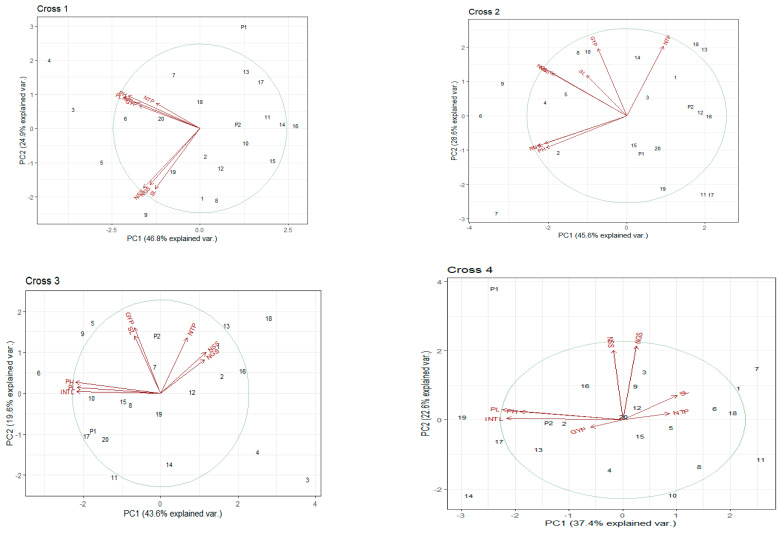

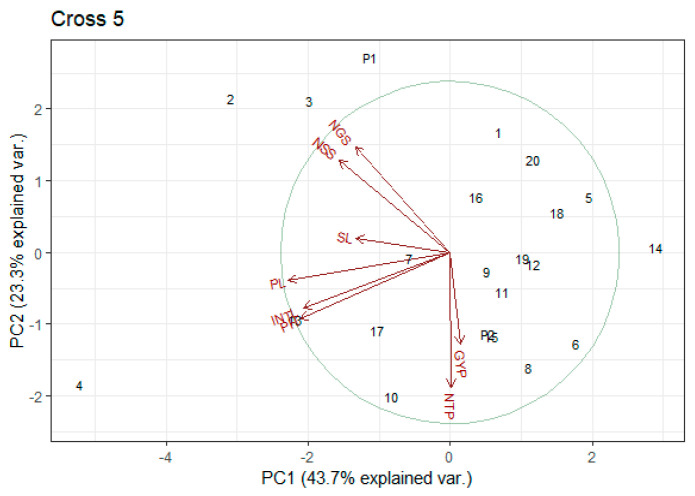
Biplot analysis for yield-contributing traits viz. plant height (PH), peduncle length (PL), peduncle internodal length (INTL), spike length (SL), number of spikelets per spike (NSS), number of grains per spike (NGS), number of tillers per plant (NTP) and grain yield per plant (GYP) parents and selected plants of F_2_ population of five crosses shown as Cross 1 to Cross 5. Circle explains the theoretical maximum extent of the arrows, added by the default confidence interval of 68%, and arrows depict the correlation among these traits.

**Figure 8:**
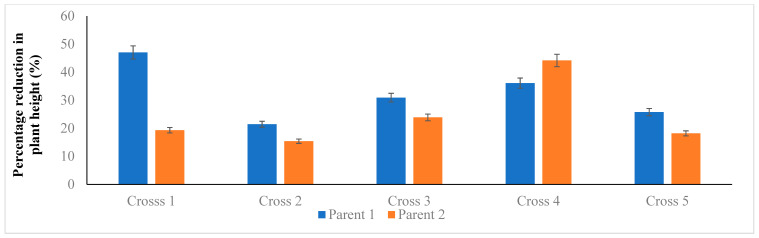
Percentage reduction in mean plant height of the segregating plants compared to their parents. This image shows percentage reduction of the plant height of plants compared to their parental genotypes.

**Figure 9:**
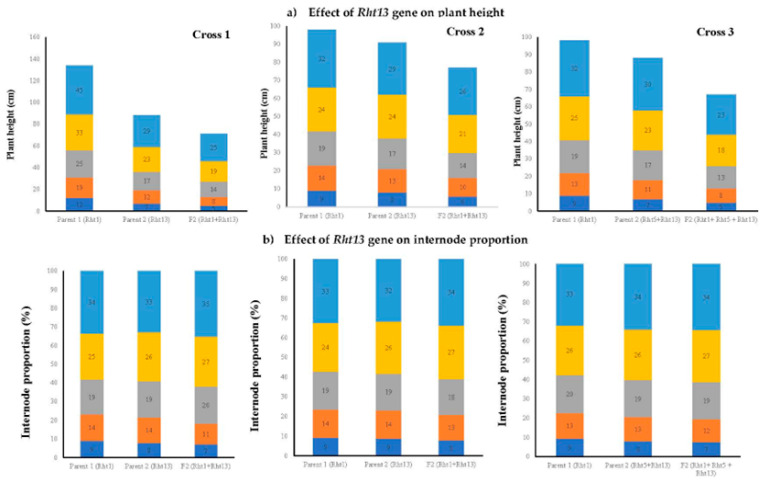
Effect of reduced plant height gene on plant height, length of internode and internode proportion in parental wheat genotypes and their F_2_ population of each Cross 1 to Cross 3 (detail of crosses is given in Table 4). (**a**) internodal length and plant height (**b**) internode proportion among parents and F_2_ plant progenies of Cross 1, 2 and 3, respectively. Each color shows different internodes from the first basal internode to the first top internode.

**Figure 10:**
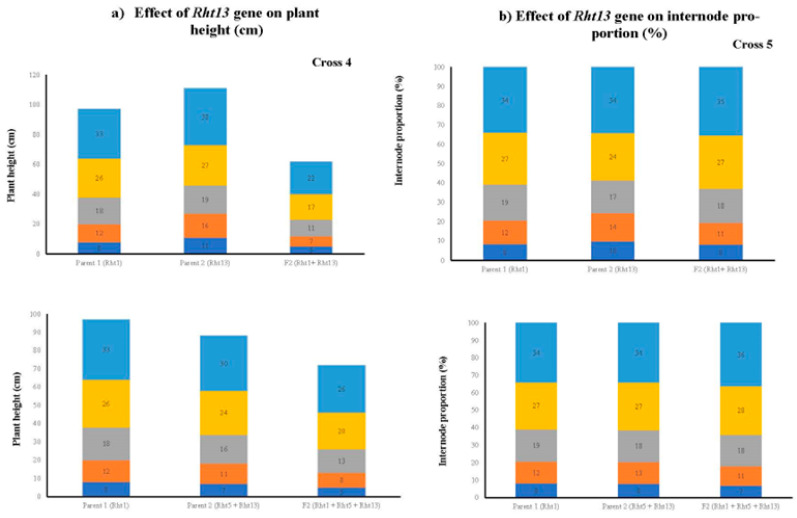
Effect of reduced height gene on plant height, length of internode and internode proportion in parental wheat genotypes and their F_2_ population Cross 4 and 5 (detail of crosses is given in Table 4). (**a**) internodal length and plant height (**b**) internode proportion among parents and F_2_ plant progenies of Cross 4 and 5, respectively. Each color shows different internodes from the first basal internode to the first top internode.

Five selected F_2_ populations were space planted with a plant-to-plant distance of 10 cm in the field, and at physiological maturity, the data were recorded for various morphological traits. The principal component biplot analysis revealed significant variability among the parental lines and the segregating populations. We recorded that the segregating populations having the GA-sensitive *Rht13* gene alone or in combination with GA-sensitive gene *Rht5* or GA-insensitive gene *Rht1* produce shorter plant height, peduncle length, peduncle internodal length, longer spike, a greater number of grains per spike, grain weight per spike and grain yield per plant (Figure 7). The presence of the *Rht13* gene alone or in combination significantly reduced the internodal length and plant height. All the cross combinations showed a variable reduction in plant height. The maximum reduction in mean plant height (47%) was recorded from the segregating population of cross 1 (PBW65/2*PASTOR × EBW01 TALL#1/SILVERSTAR-*Rht13*B//ROLF07) compared to Parent 1 (PBW65/2*PASTOR) and 19% reduction in plant height compared to arent 2 (EBW01 TALL#1/SILVERSTAR-*Rht13*B//ROLF07). The minimum reduction in plant height of 15% was recorded from Cross 2 (MILAN/S87230//BAV92/3/AKURI#1/4/MILAN × MARA) compared to Parent 2 (MARA) (Figure 8). The height of the plant having the *Rht13* gene alone or in combination with *Rht5* or *Rht1* was 17 to 40 cm shorter than its parental genotypes (Figure 9 and Figure 10; Table 13). The number of internodes varied from five to six, but most of the plants showed five internodes. We observed the maximum difference in the height reduction of the first and second uppermost internodes; the average reduction in internodal length was 8 to 12 cm compared to the mean value of both parents (Figure 9 and Figure 10). The percental difference between the first and second internode of *Rht13* carrier plants was 50% and 55%, respectively. The plant having *Rht13* reduced plant height (−32%), peduncle length (−33%) and peduncle internodal length (−26%), while it increased the number of spikelets per spike (+11%), number of grains per spike (+17%), number of tillers per plant (+88%) and grain yield per plant (+21%) compared to the plants having *Rht1*. The plants having the *Rht13* gene in combination with *Rht5* reduced plant height (−45%), peduncle length (−53%), peduncle internodal length (−48%), increased spike length (+50%), number of spikelets per spike (+32%), number of grains per spike (+19%), number of tillers per plant (+50%) and grain yield per plant (+50%) compared to the plants having *Rht1* genes (Table 13). We conclude from this experiment that the *Rht13* gene alone or in combination with other GA-sensitive genes has the potential to reduce plant height and increase grain yield per plant.

## 4. Discussion

In wheat breeding, dwarfing has been focused on the increased use of insensitive genes, especially increased fertilizer use. The dwarfing genes *Rht1, Rht2 and Rht8* were extensively used in the whole world to produce short-statured plants, reduce lodging and increase grain yield and harvest index. The effect of *Rht* genes varies across the environments. It was observed that GA-insensitive *Rht* genes (*Rht1* and *Rht2*) reduced plant height by up to 20–25% compared with wild type [17]. The GA-sensitive dwarfing genes *Rht4, Rht8, Rht9, Rht12* and *Rht13* reduced plant height by 12 to 50% with a minute or no effect on coleoptile length [18]. Extreme dwarfism was associated with reduced photosynthetic active radiation interception, harvest index, increased ground biomass and weed infestation [19]. In order to understand the effect of the GA-sensitive dwarfing gene F_1_, crosses were developed having *Rht13* genes by crossing different parental lines. It was observed that the genotypes having *Rht13* showed a significant reduction in the plant height, lodging and increased yield, agreeing with the findings of Divashuk [20]. Contrary to that, it was also observed that GA-sensitive gene *Rht18* reduced plant height (−24%) and lodging (−51%) compared to the wild-type tall plants. The reduced plant height showed an association with increased grain number (+21%), spikes number (+7%), and grain yield (+16%) [21]. The parents and crosses were evaluated in the field under normal and drought stress to estimate the effect of *Rht* dwarfing genes on the plant height, lodging % and grain yield. The results showed that *Rht13* exhibited a significant effect on plant height reduction and grain yield improvement either alone or in combination with *Rht1.* The results showed significant differences among the parents, crosses, parents vs. crosses, lines tester and interaction (lines × tester) for most of the studied traits. It was observed that the crosses having GA-sensitive *Rht* genes showed a significant reduction in plant height, lodging percentage, increased grain number and grain yield than the parents. In this study, there was a significant reduction in peduncle length, internodal length and plant height, and the genotypes with reduced peduncle length performed better under drought stress. The peduncle length was suggested as a useful indicator for the plant yield under drought stress [22]. The reduction in peduncle length due to the effect of the *Rht13* gene showed increased grain number and grain yield and showed drought tolerance due to increase water use efficiency [18]. So, the plant with reduced plant height performed better than the tall parental lines.

The genotypes having the *Rht13* gene showed increased grains/spike, grain yield, chlorophyll contents and stomatal conductance. It was observed that the assimilates of the flag leaf are the main contributor to the accumulation of dry weight in the grains [23]. High chlorophyll contents and photosynthesis rate are associated with increased grain yield [24]. The contribution of flag leaf is more during the grain filling time and grain yield as it is the main source of photosynthates at the later crop growth stages. The GA-sensitive *Rht13* gene is associated with increased stomatal conductance, increased flag leaf area and photosynthesis [18]. The leaf chlorophyll contents and stomatal conductance were higher in the parents and crosses with GA-sensitive *Rht* genes. So, these genes significantly reduced the lodging and enhanced grain yield under drought stress.

The results also showed that the presence of GA-sensitive *Rht* genes is also associated with the increased number of grains/spike, tillers/plant and grain yield. The GA-insensitive dwarfing genes *Rht1* and *Rht2* is commonly associated with a greater number of grains/spike and increased grains/unit area [25], while GA-sensitive dwarfing genes *Rht4, Rht12* and *Rht13* also showed an increased number of grains, while the *Rht8* showed a lesser effect on the number of grains [24]. *Rht13* has no significant effect on the 1000-grain weight, while *Rht8* showed a significantly increased 1000-grain weight [26]. It was also observed that the *Rht13* gene results in increased grain number and grain yield under reduced irrigation [20]. The correlation estimates showed that the plant height had a significant positive correlation with the peduncle length and internodal length, while plant height showed a negative correlation with grain yield, chlorophyll contents and stomatal conductance both under normal conditions and drought. Our results also showed that there was a significant increase in 1000-grain weight and tillers per plant in the crosses and the genotypes with *Rht13* compared with the genotypes with no *Rht13*. The genotypes also produced satisfactory yield and biomass under drought stress. It was reported that the GA-sensitive *Rht* genes showed a significant increase in grain yield and biomass, but the *Rht8* showed a slight decrease in the harvest index, grain yield and biological yield [24]. These results demonstrated that both *Rht13* alone or in combination with *Rht1* reduced the plant height, lodging (%), increased tillers/plant, spike length, grains/spike, 1000-grain weight, grain yield, chlorophyll contents and stomatal conductance.

It was also observed that the expression of GA-sensitive gene *Rht13* was enhanced under drought stress, and the enhanced expression resulted in reduced plant height, lodging and enhanced grain yield under drought stress. The reduction in plant height associated with *Rht13* was repeatable over environments and seasons. Our results showed no effect of *Rht13* on the grain yield [27]. Similarly, Cai et al. [28] observed that the upregulation of GA-sensitive and insensitive genes controls the signal transduction of signal transduction genes, cell wall structure-related genes and reactive oxygen-related genes, resulting in abiotic stress tolerance in wheat. The *Rht13* alleles were associated with an increased number of spikes, spike length, number of grains and grain yield with reduced plant height, peduncle length and internodal length. It is usually evident that height-reducing genes were associated with increased harvest index and total biomass [20,29].

## 5. Conclusions

The use of *Rht13* dwarfing genes to reduce plant height and increase grain number and grain yield was not previously reported. Selection of the genotypes and segregating populations having *Rht13* genes increases the grains/spike and harvest index compared to other GA-insensitive and GA-sensitive *Rht* genes. The increase in the number of grains showed an increased spike length and increased aerial biomass without compromising the grains’ number/ear. The presence of the *Rht13* gene in the wheat B-genome [30] and its linkage with the molecular markers may aid in the selection of this gene in the bread and durum wheat populations and enhance the adaptations to the variety of environments (drought). Further studies are needed to confirm the effect of these alleles under the disease infestation.

## 6. Patents

There is no current patent in this research.

## Figures and Tables

**Table 1:** List of parental lines having GA-sensitive and GA-insensitive *Rht* genes for hybridization in Line × Tester mating design.

Sr. No.	Lines (L)	Genes	Testers (T)	Genes
1.	Chinese Spring	*Rht13*	EBW01 TALL#1/JANZ-Rth5//NAVJ07	*Rht1* + *Rht5* + *Rht13*
2.	A-131 (27HTN/1-54)	*Rht1*	MARA	*Rht13*
3.	PBW65/2*PASTOR	*Rht1*	KINGBIRD	
4.	MILAN/S87230//BAV92*2/3/AKURI #1	*Rht1*	MAGNIF 41 ERT1	*Rht13*
5.	MILAN/S87230//BAV92/3/AKURI#1/4/MILAN	*Rht1*	EBW01 TALL#1/SILVERSTAR-*Rht*13B//ROLF07	*Rht1* + *Rht13b*
6.	AARI-11 (Shalimar 88/V 90A204//MH97)	*Rht1*		
7.	ZINCOL-16 (OASIS/SKAUZ//4*BCN/3/2*PASTOR/4/T.SPELTA PI348449/5/BACEU#1/6/WBLL1*2/CHAPIO)	*Rht1*		
8.	UJALA-16 (KIRITATATI/4/2*WEAVER/TSC/WEAVER/3/WEAVER)	*Rht1* + *Rht22*		

**Table 2:** List of crosses developed by hybridizing parental genotypes in Line × Tester mating design.

Cross No.	Cross Name	Cross No.	Cross Name
C1	L1 × T1	C21	L5 × T1
C2	L1 × T2	C22	L5 × T2
C3	L1 × T3	C23	L5 × T3
C4	L1 × T4	C24	L5 × T4
C5	L1 × T5	C25	L5 × T5
C6	L2 × T1	C26	L6 × T1
C7	L2 × T2	C27	L6 × T2
C8	L2 × T3	C28	L6 × T3
C9	L2 × T4	C29	L6 × T4
C10	L2 × T5	C30	L6 × T5
C11	L3 × T1	C31	L7 × T1
C12	L3 × T2	C32	L7 × T2
C13	L3 × T3	C33	L7 × T3
C14	L3 × T4	C34	L7 × T4
C15	L3 × T5	C35	L7 × T5
C16	L4 × T1	C36	L8 × T1
C17	L4 × T2	C37	L8 × T2
C18	L4 × T3	C38	L8 × T3
C19	L4 × T4	C39	L8 × T4
C20	L4 × T5	C40	L8 × T5

**Table 3:** List of GA-insensitive (*Rht1*) and GA-sensitive (*Rht13*) genes with internal control UBQ5 Primers.

Locus	Gene	5′F	5′R	Fragment Length (bp)	Annealing Temperature (°C)
Traes_7BS_54E859139.1 (primary)	*Rht1*	GAGAAGGTCCTGGGCACCGT	AACAGCTGCCCCCCGATGAGA	228	65
Xwms577-7B	*Rht13*	ATGGCATAATTTGGTGAAATTG	TGTTTCAAGCCCAACTTCTATT	1089	55
XM_015783851.1NCBI	UBQ5	ATGCAGATCTTCGTGAAGACC	CTAGGCCTTCTGGTTGTAGA	250	57

**Table 4:** List of parental genotypes F_2_ populations developed by crossing genotypes having GA-insensitive (*Rht1*) and GA-sensitive (*Rht13*) genes in Line × Tester mating design.

Genotype	Name	Gene
Parent 1	PBW65/2*PASTOR	*Rht1*
Parent 2	EBW01 TALL#1/SILVERSTAR-*Rht*13B//ROLF07	*Rht1 + Rht13*
Cross 1 (F_2_)	PBW65/2*PASTOR × EBW01 TALL#1/SILVERSTAR-*Rht*13B//ROLF07	*Rht1 + Rht13*
Parent 1	MILAN/S87230//BAV92/3/AKURI#1/4/MILAN	*Rht1*
Parent 2	MARA	*Rht13*
Cross 2 (F_2_)	MILAN/S87230//BAV92/3/AKURI#1/4/MILAN × MARA	*Rht1 + Rht13*
Parent 1	PBW65/2*PASTOR	*Rht1*
Parent 2	EBW01 TALL#1/JANZ-Rth5//NAVJ07	*Rht1 + Rht5 + Rht13*
Cross 3 (F_2_)	PBW65/2*PASTOR × EBW01 TALL#1/JANZ-Rth5//NAVJ07	*Rht1 + Rht5 + Rht13*
Parent 1	A-131(27-HTN/1-54)	*Rht1*
Parent 2	MAGNIF 41 ERT1	*Rht13*
Cross 4 (F_2_)	A-131(27-HTN/1-54) × MAGNIF 41 ERT1	*Rht1 + Rht13*
Parent 1	A-131(27-HTN/1-54	*Rht1*
Parent 2	EBW01 TALL#1/SILVERSTAR-*Rht*13B//ROLF07	*Rht1 + Rht13*
Cross 5 (F_2_)	A-131(27-HTN/1-54)×EBW01 TALL#1/SILVERSTAR-*Rht*13B//ROLF07	*Rht1 + Rht13*

**Table 5:** Mean square for days to heading (DTH), lodging % (LOD), plant height (PH), peduncle length (PL), internodal length (INTL), stem diameter (SD), days to maturity (DTM), spike length (SL), number of grains/spike (NGS), 1000-grain weight (TGW), grain yield per plant (GYP), chlorophyll contents (CC) and stomatal conductance (SC) of parents and F_1_ under normal conditions.

Source	DF	DTH	LOD	PH	PL	INTL	SD	DTM	SL	NGS	TGW	GYP	CC	SC
Rep. (R)	2	2.68 ^NS^	8.17 ^NS^	0.39 ^NS^	2.11 ^NS^	0.58 ^NS^	0.08 ^NS^	5.11 ^NS^	1.89 *	10.19 *	6.49 **	3.51 ^NS^	0.19 ^NS^	7.53 ^NS^
Trt. (T)	52	47.03 **	266.62 **	932.09 **	213.42 **	43.35 **	1.33 **	38.15 **	11.58 **	150.75 **	50.61 **	231.38 **	14.25 **	6095.28 **
Parents (P)	12	62.30 **	710.26 **	1105.08 **	316.34 **	39.08 **	1.95 **	38.56 **	10.91 **	134.43 **	47.76 **	285.92 **	16.26 **	8372.44 **
P vs. C	1	62.65 **	4528.57 **	3194.18 **	172.82 **	78.95 **	0.07 ^NS^	109.79 **	52.27 **	736.23 **	52.68 **	724.18 **	39.14 **	10,494.14 **
Crosses (C)	39	41.93 **	20.83 **	820.86 **	182.78 **	43.76 **	1.17 **	36.19 **	10.74 **	140.76 **	51.43 **	201.97 **	12.99 **	5281.83 **
Lines (L)	7	89.48 *	20.83 ^NS^	186.71 ^NS^	163.90 ^NS^	10.90 ^NS^	1.94 ^NS^	48.96 ^NS^	12.83 ^NS^	111.50 ^NS^	112.17 *	403.82 *	7.25 ^NS^	9784.27 *
Tester (T)	4	43.40 ^NS^	20.83 ^NS^	868.158 ^NS^	284.43 ^NS^	37.35 ^NS^	0.56 ^NS^	10.65 ^NS^	29.92 **	374.57 *	52.07 ^NS^	138.98 ^NS^	4.10 ^NS^	4569.84 ^NS^
L × T	28	29.83 **	20.83 **	972.65 **	172.98 **	52.88 **	1.06 **	36.65 **	7.48 **	114.67 **	36.16 **	160.49 **	15.69 **	4257.94 **
Error	104	2.51	3.05	2.87	2.29	1.33	0.05	2.73	0.59	2.93	1.01	1.49	0.37	30.84
Total	158													
CV														

** shows significant differences at *p* ≤ 0.01 probability level, * is significant differences at *p* ≤ 0.05 probability level and ^NS^ shows non-significant at *p* > 0.05 probability level.

**Table 6:** Mean square for days to heading (DTH), lodging % (LOD), plant height (PH), peduncle length (PL), internodal length (INTL), stem diameter (SD), days to maturity (DTM), spike length (SL), number of grains/spike (NGS), 1000-grain weight (TGW), grain yield per plant (GYP), chlorophyll contents (CC) and stomatal conductance (SC) of parents and F_1_ crosses under drought stress.

Source	DF	DTH	LOD	PH	PL	INTL	SD	DTM	SL	NGS	TGW	GYP	CC	SC
Rep. (R)	2	49.38 **	11.95 ^NS^	0.27 ^NS^	1.94 ^NS^	3.48 ^NS^	0.03 ^NS^	37.44 **	8.83 **	1.89 ^NS^	10.03 *	0.29 ^NS^	0.41 ^NS^	385.31 ^NS^
Trt. (T)	52	33.72 **	166.32 **	935.63 **	166.26 **	32.17 **	1.17 **	23.84 **	10.44 **	143.70 **	32.32 **	217.76 **	13.85 **	5212.75 ^NS^
Parents (P)	12	23.55 **	435.89 **	1159.47 **	244.67 **	25.83 **	1.77 **	29.39 **	9.77 **	149.86 **	35.20 **	271.57 **	15.58 **	7506.50 **
P vs. C	1	20.91 ^NS^	2898.29 **	2725.33 **	67.71 **	74.57 **	0.04 ^NS^	14.77 ^NS^	49.74 **	611.76 **	36.96 **	772.16 **	30.07 **	7836.31 **
Crosses (C)	39	37.17 **	520.00 **	820.87 **	144.66 **	33.03 **	1.02 **	22.36 **	9.64 **	129.81 **	31.31 **	186.98 **	12.89 **	4439.71 **
Lines (L)	7	89.23 **	13.33 ^NS^	186.71 ^NS^	150.93 ^NS^	12.10 ^NS^	1.62 ^NS^	20.49 ^NS^	12.08 ^NS^	123.31 ^NS^	50.15 ^NS^	388.81 *	7.82 ^NS^	9281.98 **
Tester (T)	4	66.94 *	13.33 ^NS^	868.16 ^NS^	254.39 ^NS^	36.57 ^NS^	0.54 ^NS^	12.65 ^NS^	27.43 **	360.19 *	34.90 ^NS^	137.75 ^NS^	5.16 ^NS^	4141.80 ^NS^
L × T	28	19.91 **	13.33 **	972.65 **	127.42 **	37.76 **	0.93 **	24.22 **	6.30 **	98.52 **	26.09 **	143.56 **	15.27 **	3271.69 **
Error	104	9.83	4.89	2.59	3.98	1.80	0.05	4.14	0.85	5.81	2.56	2.46	0.81	377.03
Total	158													

** shows highly significant differences at *p* ≤ 0.01 probability level, * is significant differences at *p* ≤ 0.05 probability level and ^NS^ shows non-significant at *p* > 0.05 probability level.

**Table 7:** GCA estimates and proportional contribution for lines (L) and testers (T) under normal condition.

GCA Effects	Morphological Traits											Physiological Traits	
	DTH	LOD	PH	PL	INTL	SD	DTM	SL	NGS	TGW	GYP	CC	SC
Lines													
L1	5.23	2.92	1.92	1.70	1.26	−0.10	2.13	−1.64	−0.52	2.91	−2.86	−0.88	−23.00
L2	−1.37	−0.42	−0.54	2.43	−0.34	0.18	0.39	1.56	−1.78	2.64	7.02	0.49	−23.60
L3	−0.63	−0.42	3.13	−0.70	−0.28	0.14	2.53	−0.11	−1.78	−1.43	1.04	−0.89	−17.47
L4	−1.23	−0.42	−1.01	−5.50	0.59	0.41	−0.88	−0.44	3.75	−2.49	−5.63	−0.03	10.67
L5	−0.30	−0.42	−1.74	−1.83	0.06	−0.72	−0.81	−0.04	−2.65	2.91	−4.88	0.74	36.60
L6	−2.43	−0.42	6.06	5.43	0.39	−0.19	−3.08	0.76	4.22	−0.16	−1.60	−0.18	−6.20
L7	−1.10	−0.42	−4.74	−1.97	−1.68	0.34	−0.81	−0.24	−2.32	0.11	8.36	−0.24	−15.13
L8	1.83	−0.42	−3.14	0.43	−0.01	−0.05	0.53	0.16	1.08	−4.49	−1.44	0.98	−38.13
PC Lines	38.3	17.95	4.08	16.09	4.47	29.76	24.28	21.45	14.22	39.14	35.89	10.02	33.25
S.E.	0.41	0.45	0.44	0.39	0.29	0.06	0.43	0.19	0.44	0.26	0.32	0.16	1.43
Tester													
T1	0.89	1.67	3.23	2.67	1.42	−0.17	0.83	1.61	5.45	−0.58	3.59	−0.13	5.10
T2	1.26	−0.42	4.23	2.63	0.92	0.17	0.41	−0.93	−1.55	0.30	−0.42	0.15	9.56
T3	−0.11	−0.42	−10.07	−4.83	−1.79	−0.11	−0.63	−0.73	1.49	−0.12	0.90	0.61	13.60
T4	0.14	−0.42	−1.11	−2.46	−0.38	−0.04	0.12	−0.68	−5.30	2.22	−2.75	−0.13	−8.65
T5	−2.19	−0.42	3.73	2.00	−0.17	0.15	−0.72	0.73	−0.09	−1.83	−1.31	−0.50	−19.61
PC Tester	10.62	10.26	10.85	15.96	8.76	4.98	3.02	28.58	27.29	10.38	7.06	3.25	8.87
PC Line × Tester	51.08	71.79	85.07	67.95	86.77	65.26	72.7	49.97	58.49	50.47	57.05	86.73	57.88
S.E.	0.32	0.36	0.35	0.31	0.24	0.04	0.34	0.16	0.35	0.21	0.25	0.12	1.13

Where, Days to heading (DTH), lodging % (LOD), plant height (PH), peduncle length (PL), internodal length (INTL), stem diameter (SD), days to maturity (DTM), spike length (SL), number of grains/spike (NGS), 1000-grain weight (TGW), grain yield per plant (GYP), chlorophyll contents (CC), stomatal conductance (SC), proportional contribution (PC) and standard error (S.E.) of lines and tester.

**Table 8:** GCA estimates and proportional contribution for lines (L) and testers (T) under drought stress in the field.

GCA Effects	Morphological Traits											Physiological Traits	
	DTH	LOD	PH	PL	INTL	SD	DTM	SL	NGS	TGW	GYP	CC	SC
Lines													
L1	4.73	2.33	1.99	0.88	1.53	−0.04	0.29	−1.83	0.13	1.30	−3.34	−0.88	−22.22
L2	−2.88	−0.33	−0.54	2.95	−0.41	0.19	0.23	1.37	−1.17	2.10	6.73	−0.44	−19.02
L3	−0.21	−0.33	3.13	−0.78	−0.21	0.15	2.16	−0.23	−2.40	−0.90	1.25	−0.87	−20.68
L4	−1.14	−0.33	−1.01	−4.92	0.13	0.38	−0.44	−0.17	2.87	−1.77	−5.17	−0.04	9.98
L5	0.73	−0.33	−1.74	−1.78	0.13	−0.66	−0.18	0.03	−2.87	2.17	−4.87	0.76	31.12
L6	−1.74	−0.33	6.06	5.28	0.53	−0.18	−2.04	0.77	3.87	0.37	−1.43	−0.19	−4.62
L7	−1.47	−0.33	−4.74	−2.18	−1.68	0.27	−0.44	−0.17	−2.93	−0.37	8.29	−0.33	−15.88
L8	1.99	−0.33	−3.14	0.55	−0.01	−0.11	0.43	0.23	3.00	−2.90	−1.48	1.09	41.32
P.C. Lines	43.08	17.95	4.08	18.73	6.58	28.66	16.45	23.87	17.05	28.75	37.32	10.89	37.52
S.E.	0.81	0.57	0.41	0.52	0.35	0.06	0.53	0.24	0.62	0.41	0.40	0.23	5.01
Tester													
T1	1.17	1.33	3.23	2.48	1.38	−0.17	0.88	1.51	5.62	−0.59	3.57	−0.10	5.57
T2	1.17	−0.33	4.23	2.36	0.72	0.13	0.50	−0.83	−1.84	−0.13	−0.71	0.16	7.61
T3	0.17	−0.33	−10.07	−4.73	−1.91	−0.09	−0.96	−0.74	1.12	−0.05	1.11	0.71	12.36
T4	0.38	−0.33	−1.11	−2.10	−0.16	−0.04	0.00	−0.70	−4.88	1.99	−2.54	−0.26	−4.93
T5	−2.88	−0.33	3.73	1.98	−0.03	0.18	−0.42	0.76	−0.01	−1.22	−1.42	−0.51	−20.60
P.C. Tester	18.47	10.26	10.85	18.04	11.35	5.42	5.8	19.19	28.46	11.43	7.56	4.09	9.57
P.C. Line × Tester	38.45	71.79	85.07	63.24	82.07	65.92	77.75	46.94	54.49	59.82	55.12	85.01	52.91
S.E.	0.64	0.45	0.33	0.41	0.27	0.05	0.42	0.19	0.49	0.33	0.32	0.18	3.96

Where, Days to heading (DTH), lodging % (LOD), plant height (PH), peduncle length (PL), internodal length (INTL), stem diameter (SD), days to maturity (DTM), spike length (SL), number of grains/spike (NGS), 1000-grain weight (TGW), grain yield per plant (GYP), chlorophyll contents (CC), stomatal conductance (SC), proportional contribution (PC) and standard error (S.E.) of lines and tester.

**Table 9:** SCA estimates and proportional contribution for lines × testers under normal conditions.

Crosses	DTH	LOD	PH	PL	INTL	SD	DTM	SL	NGS	TGW	GYP	CC	SC
L1 × T1	4.31	11.67	39.84	9.67	8.78	−0.10	−2.63	−2.61	−6.65	2.51	−9.92	−0.77	−18.83
L1 × T2	3.27	−2.92	10.18	−1.49	1.28	−0.25	−1.21	1.26	0.02	1.30	−5.80	2.57	−2.96
L1 × T3	−5.69	−2.92	−13.87	−1.70	−3.68	−0.09	2.83	0.73	2.31	−0.62	−5.43	1.74	30.33
L1 × T4	3.06	−2.92	−10.83	−4.08	0.58	0.78	4.08	0.02	4.77	−6.95	9.44	−1.31	−4.75
L1 × T5	−4.94	−2.92	−25.33	−2.20	−6.97	−0.42	−3.08	0.60	−0.44	3.76	11.71	2.23	−3.79
L2 × T1	0.58	−1.67	8.38	0040	1.38	−0.09	−2.56	0.53	4.28	−2.89	−4.93	−1.98	40.43
L2 × T2	−0.47	0.42	−24.29	−11.23	−3.12	−0.22	0.86	−2.60	−2.72	4.23	5.18	1.03	12.98
L2 × T3	−0.09	0.42	6.67	1.90	2.59	0.35	0.57	−0.14	−0.43	0.65	4.85	1.92	−27.07
L2 × T4	−1.34	0.42	12.38	12.19	0.51	0.16	0.48	0.15	0.37	−2.35	−3.79	1.13	−14.15
L2 × T5	1.33	0.42	−3.13	−3.26	−1.37	−0.21	0.65	2.07	−1.51	0.36	−1.31	−2.10	−12.19
L3 × T1	−3.16	−1.67	1.38	2.20	−0.68	−0.21	−2.03	−1.14	−3.72	0.51	8.42	−2.00	−19.03
L3 × T2	−1.87	0.42	−8.29	0.91	1.48	−0.22	-0.94	0.40	0.95	2.30	1.48	−3.39	−65.49
L3 × T3	7.84	0.42	−1.33	−5.97	1.19	−0.11	2.43	0.19	−5.76	−3.62	−0.53	−1.08	−18.53
L3 × T4	−1.08	0.42	−12.96	−11.34	−2.89	0.38	4.02	−0.85	1.37	2.38	−7.74	3.29	39.38
L3 × T5	−1.74	0.42	21.21	14.20	0.90	0.30	−3.48	1.40	7.16	−1.58	−1.64	3.17	63.68
L4 × T1	−0.89	−1.67	−12.83	−5.33	−2.21	−0.45	0.71	3.86	13.08	−0.43	−6.62	−1.61	−92.83
L4 × T2	−1.27	0.42	35.18	15.04	10.28	1.69	−1.54	−0.27	3.75	−0.96	8.19	1.83	30.38
L4 × T3	1.11	0.42	2.80	−3.17	−1.01	−0.29	2.83	−1.80	−1.29	3.78	2.39	−0.69	47.67
L4 × T4	−0.14	0.42	−19.49	2.79	−4.43	−0.18	−0.58	0.48	−4.50	−2.22	1.22	−0.34	18.92
L4 × T5	1.19	0.42	−5.66	−9.33	−2.63	−0.77	−1.42	−2.27	−11.04	−0.18	−2.73	0.82	−4.13
L5 × T1	−1.16	−1.67	−5.76	1.67	−0.68	0.49	1.64	−0.88	−4.85	−0.49	−2.17	2.11	17.90
L5 × T2	3.13	0.42	−22.09	−6.63	−5.85	0.01	2.73	−0.67	−1.85	0.30	−1.68	−3.00	0.11
L5 × T3	−0.83	0.42	−7.47	1.83	−1.42	0.11	−3.90	0.46	−1.23	2.72	−0.64	0.43	22.73
L5 × T4	−0.74	0.42	10.58	−7.54	0.78	−0.14	−3.98	−0.58	0.57	3.38	−2.79	−0.57	−6.68
L5 × T5	−0.41	0.42	24.74	10.67	6.90	−0.47	3.52	1.67	7.36	−5.91	7.28	1.04	−34.06
L6 × T1	−1.69	−1.67	−8.56	−6.60	−0.68	0.61	−1.75	1.65	6.28	−3.09	14.87	4.55	77.37
L6 × T2	−2.73	0.42	−1.89	3.11	−3.18	0.09	−1.01	−0.13	−3.38	−1.97	−7.14	−1.95	7.91
L6 × T3	0.31	0.42	5.07	3.57	−0.14	0.07	−2.97	−0.01	−6.43	4.78	−11.89	−2.21	−47.13
L6 × T4	2.06	0.42	5.11	3.19	0.78	−0.71	2.62	−1.05	−1.63	3.12	5.78	−1.79	−15.22
L6 × T5	2.06	0.42	0.28	−3.27	3.96	−0.06	3.12	−0.47	5.16	−2.84	−1.63	1.39	−22.93
L7 × T1	−0.69	−1.67	−21.43	−1.87	−4.95	−0.73	0.98	−0.01	−10.85	2.98	−3.67	−0.97	5.97
L7 × T2	−3.73	0.42	11.24	−0.16	−0.78	−0.44	0.06	0.53	−1.85	−3.23	1.82	2.28	9.84
L7 × T3	0.31	0.42	−4.13	−1.37	−0.08	0.56	−2.57	0.99	11.11	−4.48	8.77	−1.62	−23.20
L7 × T4	0.73	0.42	2.58	−0.08	1.84	−0.27	−5.32	0.62	1.23	1.85	−3.98	−0.46	−12.28
L7 × T5	3.39	0.42	11.74	3.47	3.97	0.89	6.85	−2.13	0.36	2.89	−2.94	0.76	19.68
L8 × T1	2.71	−1.67	−1.03	0.07	−0.95	0.39	5.64	−1.41	2.42	0.91	4.01	0.67	−10.97
L8 × T2	3.67	0.42	−0.03	0.44	−0.12	−0.68	1.06	1.46	5.08	−1.97	−2.05	0.63	7.24
L8 × T3	−2.96	0.42	12.27	4.90	2.26	−0.58	0.77	−0.41	1.71	−3.22	2.48	1.52	15.20
L8 × T4	−2.54	0.42	12.65	4.85	2.84	0.13	−1.32	1.22	−2.17	0.78	4.31	0.03	−5.22
L8 × T5	−0.88	0.42	−23.86	−10.27	−4.03	0.73	−6.15	−0.87	−7.04	3.49	−8.75	−2.85	−6.26
PC L × T	51.08	71.79	85.07	67.95	86.77	65.26	72.7	49.97	58.49	50.47	57.05	86.73	57.88
S.E.	0.92	1.01	0.98	0.87	0.67	0.13	0.95	0.44	0.99	0.58	0.71	0.5	3.21

Where, Days to heading (DTH), lodging % (LOD), plant height (PH), peduncle length (PL), internodal length (INTL), stem diameter (SD), days to maturity (DTM), spike length (SL), number of grains/spike (NGS), 1000-grain weight (TGW), grain yield per plant (GYP), chlorophyll contents (CC), stomatal conductance (SC), proportional contribution of lines × tester (PC L×T) and standard error (S.E.).

**Table 10:** SCA estimates and proportional contribution for lines × testers for lines × testers under drought stress in the field.

Crosses	DTH	LOD	PH	PL	INTL	SD	DTM	SL	NGS	TGW	GYP	CC	SC
L1 × T1	3.90	9.33	39.84	7.45	7.68	−0.001	−2.88	−2.71	−5.55	0.99	−9.21	−1.06	−26.70
L1 × T2	1.90	−2.33	10.18	−2.09	1.35	−0.26	−0.83	0.96	0.91	0.53	−5.26	2.65	−0.41
L1 × T3	−4.77	−2.33	−13.87	−0.34	−3.69	−0.11	2.63	0.54	1.95	−0.88	−5.39	1.44	40.17
L1 × T4	0.69	−2.33	−10.83	−3.30	0.56	0.77	3.67	0.17	3.62	−4.59	9.29	−0.75	−10.20
L1 × T5	−1.73	−2.33	−25.33	−1.72	−5.90	−0.41	−2.58	1.04	−0.93	3.95	10.57	−2.94	−2.87
L2 × T1	0.83	−1.33	8.38	−0.28	0.62	−0.01	−3.48	1.09	4.25	−2.14	−5.03	−1.63	44.42
L2 × T2	0.17	0.33	−24.29	−9.83	−2.72	−0.23	1.57	−2.58	−2.96	4.40	5.75	0.19	11.73
L2 × T3	2.83	0.33	6.67	1.59	2.58	0.37	0.03	−0.33	0.42	0.32	4.70	2.37	−26.69
L2 × T4	0.96	0.33	12.38	10.63	0.49	0.08	−0.27	0.30	0.08	−2.73	−3.91	1.05	−20.07
L2 × T5	−4.79	0.33	−3.13	−2.12	−0.97	−0.21	2.15	1.51	−1.79	0.15	−1.51	−1.97	−9.40
L3 × T1	−1.50	−1.33	1.38	2.12	−0.92	−0.11	0.93	−1.31	−4.02	1.19	8.19	−1.79	−17.56
L3 × T2	−1.83	0.33	−8.29	0.91	1.42	−0.30	−1.70	0.69	2.44	2.07	1.33	−3.22	−51.28
L3 × T3	4.83	0.33	−1.33	−5.01	1.04	−0.14	0.43	−0.06	−4.52	−3.68	−0.55	−1.06	−16.36
L3 × T4	−1.38	0.33	−12.96	−9.63	−2.38	0.25	2.47	−0.43	−0.52	1.94	−7.19	3.08	44.93
L3 × T5	−0.13	0.33	21.21	11.62	0.83	0.31	−2.12	1.11	6.61	−1.52	−1.79	2.99	40.27
L4 × T1	−0.23	−1.33	−12.83	−4.75	−1.558	−0.44	1.86	3.63	12.72	−1.28	−5.69	−1.79	−55.90
L4 × T2	−1.23	0.33	35.18	13.38	8.42	1.63	0.23	−0.04	2.51	−0.73	7.38	2.25	−4.61
L4 × T3	−0.23	0.33	2.80	−2.21	−0.96	−0.28	1.69	−1.46	−0.78	3.52	1.79	−0.56	39.64
L4 × T4	−0.78	0.33	−19.49	2.17	−4.04	−0.17	−1.93	−0.17	−4.78	−0.86	−0.88	−0.32	24.27
L4 × T5	2.48	0.33	−5.66	−8.58	−1.83	−0.74	−1.85	−1.96	−9.66	−0.65	−2.61	0.42	−3.40
L5 × T1	−1.77	−1.33	−5.76	1.45	−0.92	0.50	0.59	−0.91	−4.55	0.46	−2.85	1.96	−13.03
L5 × T2	2.23	0.33	−22.09	−5.09	−4.92	−0.01	2.30	−0.58	−2.43	−1.67	−2.05	−2.86	7.93
L5 × T3	0.23	0.33	−7.47	1.33	−0.29	0.01	−2.91	0.68	−0.38	2.25	−0.59	0.58	22.18
L5 × T4	−1.64	0.33	10.58	−7.30	0.29	−0.11	−3.53	−0.37	1.28	3.88	−2.03	−0.93	6.48
L5 × T5	0.94	0.33	24.74	9.62	5.83	−0.39	3.55	1.18	6.08	−4.92	7.53	1.26	−23.53
L6 × T1	−0.63	−1.33	−8.56	−4.95	0.02	0.51	−1.88	1.69	6.05	−2.74	14.16	4.45	75.37
L6 × T2	−1.97	0.33	−1.89	1.51	−2.65	0.04	−1.17	−0.31	−2.15	−1.87	−6.87	−1.98	19.65
L6 × T3	0.03	0.33	5.07	2.59	−0.35	0.16	−2.04	−0.06	−7.12	4.38	−11.03	−1.96	−48.43
L6 × T4	1.16	0.33	5.11	4.30	0.89	−0.55	3.67	−0.77	−1.12	2.34	4.95	−1.48	−22.13
L6 × T5	1.41	0.33	0.28	−3.45	2.10	−0.15	1.42	−0.56	4.34	−2.12	−1.21	0.97	−24.47
L7 × T1	−1.90	−1.33	−21.43	−1.15	−3.78	−0.75	0.53	−0.04	−11.15	1.99	−2.72	−1.24	3.30
L7 × T2	−2.90	0.33	11.24	−0.69	−0.78	−0.27	−0.77	0.63	−1.03	−1.13	1.39	2.75	14.26
L7 × T3	−0.23	0.33	−4.13	−0.94	0.18	0.46	−1.31	0.54	11.35	−4.22	8.92	−2.25	−19.49
L7 × T4	2.23	0.33	2.78	−0.57	1.09	−0.26	−2.93	0.83	0.68	0.74	−4.32	−0.44	−21.53
L7 × T5	2.81	0.33	11.74	3.35	3.30	0.82	4.48	−1.96	0.14	2.62	−3.27	1.18	23.47
L8 × T1	1.30	−1.33	−1.03	0.12	−1.12	0.30	4.33	−1.44	2.25	1.53	3.14	1.11	−9.90
L8 × T2	3.63	0.33	−0.03	1.91	−0.12	−0.59	0.37	1.23	2.71	−1.60	−1.66	0.23	2.73
L8 × T3	−2.70	0.33	12.27	2.99	1.51	−0.47	1.49	0.14	−0.92	−1.68	2.14	1.43	8.98
L8 × T4	−1.24	0.33	12.64	3.70	3.09	−0.001	−1.13	0.43	0.75	−0.73	4.11	−0.21	−1.73
L8 × T5	−0.99	0.33	−23.86	−8.72	−3.37	0.77	−5.05	−0.36	−4.79	2.48	−7.72	−2.55	−0.07
PC L × T	38.45	71.79	85.07	63.24	82.07	65.92	77.75	46.94	54.49	59.82	55.12	85.01	52.91
S.E.	1.81	1.28	0.93	1.15	0.78	0.13	1.18	0.53	1.39	0.92	0.91	0.52	11.21

Where, days to heading (DTH), lodging % (LOD), plant height (PH), peduncle length (PL), internodal length (INTL), stem diameter (SD), days to maturity (DTM), spike length (SL), number of grains/spike (NGS), 1000-grain weight (TGW), grain yield per plant (GYP), chlorophyll contents (CC), stomatal conductance (SC), proportional contribution of lines × tester (PC L×T) and standard error (S.E.).

**Table 11:** Genetic components for days to heading (DTH), lodging % (LOD), plant height (PH), peduncle length (PL), internodal length (INTL), stem diameter (SD), days to maturity (DTM), spike length (SL), number of grains/spike (NGS), 1000-grain weight (TGW), grain yield per plant (GYP), chlorophyll contents (CC) and stomatal conductance (SC) of wheat genotypes under normal conditions in the field.

Genetic Components	Morphological Traits											Physiological Traits	
	DTH	LOD	PH	PL	INTL	SD	DTM	SL	NGS	TGW	GYP	CC	SC
Cov H.S. (lines)	3.97	2.37 × 10^−15^	−52.39	−0.61	−2.79	0.06	0.82	0.36	−0.21	5.07	16.22	−0.56	368.42
Cov H.S. (tester)	0.56	1.63 × 10^−15^	−4.35	4.64	−0.65	−0.02	−1.08	0.94	10.83	0.66	−0.89	−0.48	12.99
Cov H.S. (average)	0.21	1.94 × 10^−16^	−2.68	0.17	−0.16	0.001	−0.01	0.06	0.46	0.27	0.73	−0.05	18.06
Cov F.S. (average)	17.24	5.93	224.33	68.27	10.79	0.38	9.79	5.39	65.77	21.93	77.64	2.88	2057.73
σ ^2^ gca	0.43	3.89 × 10^−16^	−5.35	0.35	−0.32	0.003	−0.02	0.12	0.92	0.54	1.46	−0.09	36.12
σ ^2^ sca	9.11	5.93	323.26	56.89	17.18	0.34	11.31	2.29	37.25	11.72	53.00	5.11	1409.03
σ ^2^ gca/σ ^2^ sca	0.05	6.5 × 10^−17^	−0.02	0.01	0.02	0.001	−0.002	0.05	0.02	0.05	0.03	−0.02	0.03
F = 0 Additive genetic variance	0.85	7.78 × 10^−16^	−10.71	0.69	−0.64	0.01	−0.03	0.23	1.84	1.08	2.93	−0.19	72.24
F = 1 Additive genetic variance	0.43	3.89 × 10^−16^	−5.35	0.35	−0.32	0.003	−0.02	0.12	0.92	0.54	1.46	−0.09	36.12
F = 0 Variance due to dominance	18.21	11.86	646.52	113.79	34.37	0.68	22.62	4.59	74.49	23.43	106.00	10.22	2818.06
F = 1 Variance due to dominance	9.11	5.93	323.26	56.89	17.18	0.34	11.31	2.29	37.25	11.72	53.00	5.11	1409.03

**Table 12:** Genetic components for days to heading (DTH), lodging % (LOD), plant height (PH), peduncle length (PL), internodal length (INTL), stem diameter (SD), days to maturity (DTM), spike length (SL), number of grains/spike (NGS), 1000-grain weight (TGW), grain yield per plant (GYP), chlorophyll contents (CC) and stomatal conductance (SC) of wheat genotypes under drought stress conditions in the field.

Genetic Components	Morphological Traits											Physiological Traits	
	DTH	LOD	PH	PL	INTL	SD	DTM	SL	NGS	TGW	GYP	CC	SC
Cov H.S. (lines)	4.62	3.55 × 10^−16^	−52.39	1.57	−1.71	0.05	−0.25	0.43	1.65	1.60	17.35	−0.49	400.69
Cov H.S. (tester)	1.96	4.44 × 10^−16^	−4.35	5.29	−0.05	−0.02	−0.48	0.88	10.90	0.37	−0.24	−0.42	36.25
Cov H.S. (average)	0.30	3.54 × 10^−17^	−2.68	0.30	−0.08	0.001	−0.03	0.05	0.55	0.09	0.77	−0.04	20.60
Cov F.S. (average)	16.29	2.81	224.42	57.86	9.00	0.326	4.99	4.88	62.73	11.49	73.64	2.87	1729.38
σ ^2^ gca	0.61	7.07 × 10^−17^	−5.35	0.61	−0.17	0.003	−0.06	0.12	1.10	0.18	1.53	−0.08	41.21
σ ^2^ sca	3.36	2.811	323.35	57.86	11.98	0.294	6.69	1.82	30.90	7.84	47.03	4.82	964.89
σ ^2^ gca/σ ^2^ sca	0.18	2.51 × 10^−17^	−0.02	0.01	−0.01	0.010	−0.01	0.07	0.04	0.02	0.03	−0.02	0.04
F = 0 Additive genetic variance	1.22	1.41 × 10^−16^	−10.71	1.22	−0.33	0.006	−0.13	0.24	2.21	0.37	3.06	−0.17	82.41
F = 1 Additive genetic variance	0.61	7.07 × 10^−17^	−5.35	0.61	−0.17	0.003	−0.06	0.12	1.10	0.18	1.53	−0.08	41.21
F = 0 Variance due to dominance	6.72	5.62	646.71	82.29	23.91	0.588	13.38	3.63	61.81	15.69	94.07	9.64	1929.78
F = 1 Variance due to dominance	3.36	2.81	323.35	41.15	11.98	0.294	6.69	1.82	30.90	7.84	47.04	4.82	964.89

**Table 13:** Mean value of yield contributing traits measured among parents and F_2_ populations of the selected plants for *Rht1, Rht13, Rht5 + Rht13, Rht1 + Rht5 + Rht13* dwarfing alleles.

Traits	Mean Value of Alleles				Contrast among Alleles		
	*Rht1*	*Rht13*	*Rht5 + Rht13*	*Rht1 + Rht5 + Rht13*	*Rht1* vs. *Rht 13*	*Rht1* vs. *Rht5 + Rht13*	*Rht1* vs. *Rht1 + Rht5 + Rht13*
Plant height (PH), cm	134	91	88	74	−32	−34	−45
Peduncle length (PL), cm	45	30	31	21	−33	−31	−53
Peduncle internodal length (INTL), cm	23	17	16	12	−26	−30	−48
Spike length (SL), cm	10	12	15	15	20	30	50
Number of spikelets per spike (NSS)	19	21	19	25	11	0	32
Number of grains per spike (NGS)	54	63	68	64	17	26	19
Number of tillers per plant (NTP)	8	15	15	12	88	88	50
Grain yield per plant (GYP), g	14	17	18	21	29	29	50

## Data Availability

The data will be available on request to the corresponding author zulfiqarpbg@hotmail.com.
